# P01.07. The Chinese herbs Chuan Bei Mu and Huang Qin can produce dose-dependent biphasic response in endometrial cancer cells

**DOI:** 10.1186/1472-6882-12-S1-P7

**Published:** 2012-06-12

**Authors:** K Ahmad, J Pirog, V Syed, Y Jiang, H Nguyen

**Affiliations:** 1 Northwestern Health Sciences University, Bloomington, USA; 2 Uniformed Services University of the Health Sciences, Bethesda, USA

## Purpose

We shortlisted four commonly-used herbs from the Chinese Materia Medica, including Lian Qiao (Forsythiae Fructus), Chuan Bei Mu (Fritillariae cirrhosae Bulbus), Bai Zhi (Angelicae dahuricae Radix) and Huang Qin (Scutellariae Radix) due to suggestions in classical writings that they could possess antitumor properties.

## Methods

We were interested in testing the herbs’ cytotoxic potential and used Ishikawa endometrial cancer cells. Cells were incubated for 24 hours in varying concentrations (1-500 µg/ml).

## Results

We found that at low doses Huang Qin induced cytotoxicity (1 µg/ml), but at high doses, 50-300 µg/ml, the same herb induced cell proliferation. Chuan Bei Mu exhibited a similar biphasic response with cytoxicity at 1-200 µg/ml but proliferation at 400 µg/ml. Data on Lian Qiao was scattered but the herb induced cytotoxicity at low doses from 1-10 µg/ml and less significantly at high doses. Bai Zhi was the only herb that did not induce cytotoxicity in endometrial cancer cells.

## Conclusion

Taken together, the preliminary data underscore the effect of these herbs as bioactive compounds with chemotherapeutic potential, and contrastingly of their ability to enhance cancer cell growth or confound the effect of chemotherapeutic agents.

